# Risk assessment of ICU patients through deep learning technique: A big data approach

**DOI:** 10.7189/jogh.12.04044

**Published:** 2022-05-30

**Authors:** Xiaobing Huang, Shan Shan, Yousaf A Khan, Sultan Salem, Abdullah Mohamed, El-Awady Attia

**Affiliations:** 1 Research Center for Ageing Society of Jiangxi Provincial Association of Social Science, Gannan Normal University, Ganzhou, China; 2 School of Business Analytics and Decision Making, Coventry University, Coventry, UK; 3 Department of Mathematics and Statistics, Hazara University Mansehra, Pakistan; 4 Faculty of Economics, University of Birmingham, Birmingham, UK; 5 Research Centre, Future University in Egypt, New Cairo, Egypt; 6 Department of Industrial Engineering, College of Engineering, Prince Sattam Bin Abdulaziz University, AI Kharj, Saudi Arabia; 7 Mechnical Engineering Department, Faculty of Engineering (Shoubra), Benga University, Cairo, Egypt

## Abstract

**Background:**

Intensive Care Unit (ICU) patients are exposed to various medications, especially during infusion, and the amount of infusion drugs and the rate of their application may negatively affect their health status. A deep learning model can monitor a patient's continuous reaction to tranquillizer therapy, analyze the treatment plans of experts to avoid severe situations such as reverse medication associations, work with a convenient mediator, and change the treatment plans of specialists as needed.

**Methods:**

Generally, patients' treatment histories are linked together via a period grouping connection, which is usually burdened by missing information. Displaying time-succession via Repetitive Neural Organization (RNO) is the best available solution. However, it's possible that a patient's treatment may be prolonged, which RNN may not be able to demonstrate in this manner.

**Results:**

We propose the use of the LSTM-RNN driven by heterogeneous medicine events to predict the patient's outcome, as well as the Regular Language Handling and Gaussian Cycle, which can handle boisterous, deficient, inadequate, heterogeneous, and unevenly tested prescription records of patients while addressing the missing value issue using a piece-based Gaussian cycle.

**Conclusions:**

We emphasize the semantic relevance of every medication event and the grouping of drug events on patients in our study. We will focus specifically on LSTM-RNN and Phased LSTM-RNN for showing treatment results and information attribution using bit-based Gaussian cycles. We worked on Staged LSTM-RNN.

The enormous amount of clinical data generated both by ICU patients and staff, as well as the constant estimations of patients’ health outcomes, can provide AI calculations with an amazing opportunity. The detection approach [1] and hereditary data extraction [2] are only two examples of current clinical examinations that deeply rely on AI technologies. This creates a great opportunity for clinicians because of the growing use of Electronic Health Records (EHR). EHRs are collections of data compiled from clinical assessments of individuals. They consist of diverse information, including segment data, textual clinical notes, and clinical openings. ICU patients will benefit greatly from an AI model based on their EHR data. There are, however, several issues with EHR data, including sparseness, anomalies, heterogeneity, and turbulence in the real data. AI approaches have been utilized extensively in the past to analyze EHR data, concentrating mostly on predicting and presenting problems (for example, patient result expectation and time-series demonstration). There have been a few recent attempts at using deep learning alongside factual investigation [3-5] and AI techniques [5-8]. Compared to conventional AI, deep learning has greater performance in picture arrangement [9], discourse acknowledgement [10], normal language handling [11], and other applications. Its primary goal is to identify and extract semantically relevant information from test results. Rich data from authentic clinical records may be mined to help illuminate how learning is represented in EHR information. In the development and application of EHR. deep learning and natural language processing technology based on it are also expected to conduct feature demonstration and representation learning on patient’s medical data

The greatest challenges inherent to using EHR data are its high aspect, heterogeneity, missing observations, and reliance on long-term data, among others. EHR contains many clinical highlights from various sources (for example, important bodily function estimations, portion names, doctors' notes, depiction of clinical events, etc.), which brings about high heterogeneity of data type and aspect. Consequently, the greatest challenge is using EHR data in practice. Examined frequencies vary depending on the type of clinical event. While medication-related events are often measured in days, urgent sign-related events are typically measured in hours, and brainwave information testing is typically measured in seconds [12,13]. To avoid standardization issues, it may be necessary to review data infrequently, which may not capture all its events over an extended period.

The time-consistent nature of EHR is one of its most important features. With its memory instrument inside its cell structure, the flexible structure of a recurrent neural network (RNN) makes it the most state-of-the-art technique for modelling successive types of data. However, due to the frequency and shortness of hospitalization events, the RNN may have problems with the slope blast or evaporation inclination. Long Short-Term RNN (LSTM) was introduced by Sepp Hochreiter [14] in 1997 to demonstrate an arrangement kind of information spamming across 1000-time steps. For such long-term slack projects, LSTM can use the “dropout” instrument. LSTM's preparation cycle has been sped up by the rapid growth of EHR's data volume, which is unavoidable given EHR’s current usage. LSTM may not be the best choice considering potential future improvements to EHR. Recent research has shown that Staged LSTM can speed up preparatory interactions, especially for extended and occasion-based information grouping [15]. In any case, using Staged LSTM directly isn't beneficial when dealing with heterogeneous information structures.

EHR data consists of various clinical events and readings, many of which have a rich idle link. For example, cholesterol testing should be more closely linked to the heart/liver than the kidney. Similitudes between linked clinical events can be preserved through semantic depiction, while the distinctions between other clinical events are captured. At that point, clinical events are categorized based on their time stamp and Gaussian process (GP) is used to assign the missing attributes to each one. For text-based clinical notes, the inclusion of depiction is a crucial technique. Several new studies focused on multivariate information ascription [16]. To create the inserting space for semantic links among diverse clinical occasions, we use semantic portrayal and normal language handling innovation. One way of addressing the whole note as a lattice and concentrating data in a clinical note is the development of a word installing space. We will use Gaussian Interaction and then the deep learning models to credit any missing clinical event data in the framework's information preparation section. For the purposes of this study, we present an improved Staged LSTM that focuses on the influence of sporadic heterogeneous occasion-based long-term clinical information on the mortality of patients. As part of the evaluation, we compare the performance of the Staged LSTM with the LSTM model. In addition, we employ semantic depiction in all therapeutic settings. The specific contributions of this research are as follows:

1. It addresses the problem of heterogeneous time-series occasion data from several sources of occasional testing in electronic clinical records, we present an improved Staged LSTM. When constructing models using this framework, consideration should be given to how to deal with heterogeneous time-series events and how to handle regular language processing.
2. Using several procedures and selecting the most effective one, resolves the issue of missing data in clinical record time series (Gaussian Cycle Relapse Demonstrating).
3. We demonstrate the applicability of the proposed method on medical records by trials of mortality hazard projection using Imitate III clinical liquid-related clinical events and conclusion reports.

## Literature review

### Medical word embedding space

A good focus for this research could be patients' different experiences with several clinical prescriptions. Using the word2Vec model, it is easier to distinguish between homogeneous and heterogeneous drug events.

Various approaches to portraying medical care information are currently being researched. One approach is to provide a patient-friendly depiction of a dormant region. These pieces can be saved and modelled using the inert space representation. Summed-up Straight Unique Models (GLDM) were proposed by Caballero et al. [17] as a method of showing individuals with high mortality risk in an inert condition. Subsequently, they demonstrate that the model can predict an increase in mortality risk before it occurs. The latent state changes over time and was represented by Krompass et al [18] using a bespoke, transient, multi-faceted idle installation environment. This latent space can preserve the features of each individual patient. Using Inert Dirichlet Designation (LDA), Jonnagaddala et al. [19] construct point circulatory loads for each understanding as highlights to differentiate patient smoking status. A word2Vec model is built using biomedical examination articles and biomedical text mining as part of an ongoing exploratory effort. Using a strategy that focuses on the similarities or relationships between terms might better maintain their semantic relevance in clinical jargon.

Exploring the possibilities of diverse word depictions, several researchers have used standard language processing approaches. For example, word2Vec [20,21], GloVe [22], and fastText [23,24] have been developed recently to construct word insertion. To create the word inserting space and the word implanting space, Gokul S. Krishnan and Sowmya Kamath S. looked at three different techniques with varying boundary definitions [25-27].

Finally, the four standard grading frameworks were used to examine the word inserting space highlight representation. Skip-Gram (SG) methods and Arbitrary Timberland classifier can outperform SAPS-II, Couch, APS-III, and Desert spring by 43%-52%, according to their data. Continuing patient mortality forecast assignments have also used other NLP methodologies, such as subject demonstrating. For mining illness clinical notes, Chan et al. [28] examined the possibility of using points showing inactive space.

Subject display was used to interpret clinical literature and the link between theme demonstrating and the information available on the change board was examined. The findings by Chan et al. [28] suggest a few genotype-aggregate relationships provide useful differentiating evidence. The term “implanting space” has been used in clinical notes to describe ongoing research into patients' death forecasts. With all these unstructured clinical messages in the EHR, it is possible for a lack of vector representation or inability to handle the entire context to arise.

## Data imputation

The Gaussian Process is utilized to deal with the problem of missing values. It is possible to think of the missing value issue as a way of predicting the values that will be absent from a set of continuous quantities. Using observed data, we may build a Gaussian Process regression model and use the output to forecast the results when data are missing. There is a popular interpretation of the Gaussian Process as a distribution over functions and inference which takes place in this space of functions. It is therefore possible to estimate the missing values using the maximum likelihood technique when using a Gaussian Process regression model. In the methods section, data imputation specifics with the Gaussian Process will be covered.

In comparison to other machine learning algorithms, deep learning has been shown as an excellent strategy for predicting patient outcomes. However, standard Deep Neural Network (DNN) is unable to model past data while accounting for the data's sequential temporal dependence. Sequence data refers to data that has a temporal time dependency. Sequence data are a perfect fit for the Recurrent Neural Network. Long sequence lengths are typical in patients’ data, as is the case with long-term reliance. The RNN's backpropagation demands more calculations during training. RNN-based deep neural network models cannot be trained when the data has a long-term temporal dependency. This is mostly due to Sepp Hochreiter's [30] gradient disappearance or explosion problem. Using vanishingly tiny gradients to update weights exponentially causes the vanishing gradient issue. Long-term reliance in training data might cause this difficulty, which inhibits the model from training properly. Sepp Hochreiter's LSTM RNN adds a series of “gates” to the neuron structure [14]. There is no need for the exponentially declining component in the computation of neuron status because this collection of “gates” defines the drop-out and update information.

## METHODS

### Deep neural network

Scaling up EHR has a significant impact on the amount of data available. Patients’ data are considerably more time-dependent in the long term. In contrast, the foundation of LSTM does not fit this pattern. Neuronal research in recent years has focused on altering the RNN/LSTM structure. Thorsen-Meyer et al. [32] used a machine learning technique to study the dynamic mortality of critical care unit patients. Strand et al. [33] evaluated SAPS II and SAPS 3 in a Norwegian acute care unit. Christensen et al. [34] compared the Charlson comorbidity index to SAPS and APACHE scores to predict death in intensive care. Using recurrent neural networks, Aczon et al. [35] studied dynamic mortality risk forecasts in paediatric critical care. Kounik et al. [36] proposed the Clockwork RNN (CN-RNN). Multiple modules make up the CN-RNN hidden layer. A specified clock is assigned to each module, and each clock has a predetermined pace. Because the slower clock rate neuron is connected to faster clock rate neurons, this CN-RNN architecture helps contain knowledge from past computations by utilizing multiple clock rates. The CN-RNN works effectively in long-term time dependencies thanks to the use of a clock with a distinct clock rate. To retain the Phased-LSTM training error in backpropagation and speed up the training process for long-term sequence clinical data, Neil [15] designed an enhanced LSTM neuron called phased-LSTM, which adds a temporal gate to the LSTM to adjust the phase of the neuron. We will cover the specifics in the following sections.

### Proposed model for medical data

#### Medical event representation

A total of 53 423 adult patients (aged ≥16 years) were hospitalized in critical care units in the United States between 2001 and 2012. A total of 38 597 adult patients and 49 785 hospitalizations were included in the study. Adult patients had an average age of 65.8 years, with a male preponderance of 55.9% and an in-hospital death rate of 11.5%. An ICU stay typically lasted 2.1 days, whereas a hospital stays typically lasted 6.9 days. There were 4579 chart events and 380 laboratory events (laboratory statistics) accessible for each hospital admission on average. **Table 1** provides an in-depth look into critical care units and the services they provide.

**Table 1:** Details of the patient by critical care unit on hospital admission (aged ≥16 years)

Critical Care Unit	CCU	CSRU	MICU	SICU	TSICU	Total
Distinct patients	5674	8091	13 649	6372	4811	38 597(100%)
Hospital admissions	7258	9156	19 770	8110	5491	49 785(100%)
Distinct ICU stays	7726	9854	21 087	8891	5865	53 423(100%)
Age in years	70.1	67.6	64.9	63.6	59.9	65.8 (52.8-77.8)
Gender-male	4203	6000	10193	4251	3336	27 983 (55.9%)
ICU length of stay	2.2	2.2	2.1	2.3	2.1	2.1 (1.2-4.6)
Hospital length of stay	5.8	7.4	6.4	7.9	7.4	6.9 (4.1-11.9)
ICU mortality	685	353	2222	813	492	4565 (8.5%)
Hospital mortality	817	424	2859	1020	628	5748 (11.5%)

CCU – coronary care unit, CSRU – cardiac surgery recovery unit, MICU – medical intensive care unit, SICU – surgical intensive care unit, TSICU – trauma surgical intensive care unit

We extracted fluid-input-related event records from the MIMIC III database and the following information from this table:

- “**itemid**” is the identifier for a single measurement type,
- “**rate**” lists the rate at which the drug or substance was administered to the patient,
- “**totalamount**” lists the total amount of the fluid in the bag containing the solution,
- “**starttime**” and “**endtime**” record the start and end time of an input/output event. This information will be used to build per-patient time-series event data.

**Figure 1:**
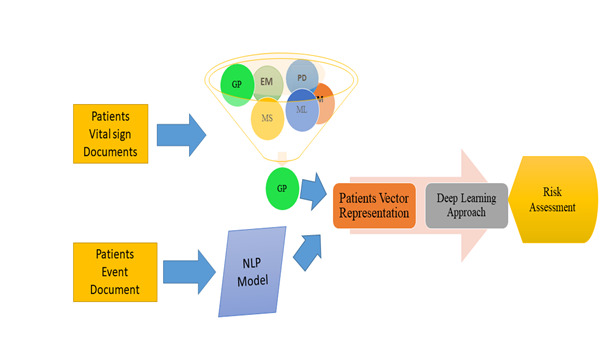
Conceptual framework for mortality risk assessment.

**Figure 1** shows our complex learning structure pipeline in action. The word vector depiction of the medication/substance is the pipeline’s information. At this stage, we increased the vector aspect by adding the remainder of the numeric value components. (occasion>, aggregate total, rate, internal heat level), where “occasion>” is the semantic word vector depiction for this drug occasion and the other vector elements are the mathematical estimation values that may be used to address each information vector. As a guideline, we used the mean value in that range for internal heat level, heart rate, breath rate, and pulse rate.

**Figure 2:**
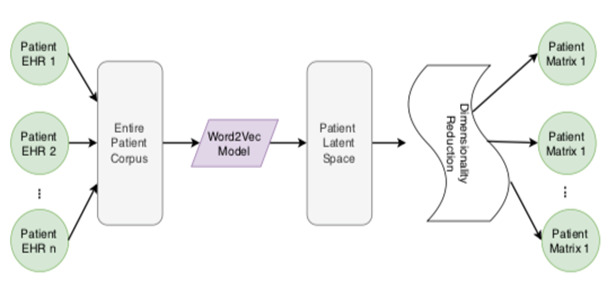
Word Vector Representation Medical Event.

**Figure 2** shows how to build up each calm's clinical occasion depiction. We treat every prescription event as if they are interlinked, and as if only the patient’s clinical situation matters. NLP word vector representations may be used for detecting this type of link, as the model should theoretically provide a place to locate the linked clinical events. For example, the anti-cardiolipin antibody is used in haematology. Heparin, another fluid medicine used in haematology linked to blood clustering, should be nearby when used in clinical settings. If the two fluid medications semantically unconnected to the therapy are mixed, a human error may be a factor. The use of semantic connections on the EHR side can help reduce clinical errors.

**Figure 3:**
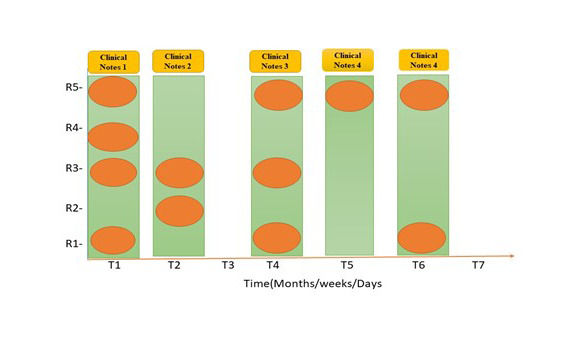
Graphical presentation of missing value problems in sample data.

We used the word2Vec neural association model to create a word vector representation. **Figure 3** shows our framework in which these “words” go into the patient's “record”, which is kept in a database. All the patients build up the entire corpus. To properly build up the word2vec model, we use two algorithms: CBOW and Skip-Gram (SG) computations. SG uses the context words to predict the target words, while CBOW uses the target word to predict the context word. There are two ways to create a word vector image. Each clinical event may be seen as a thick vector in our far-reaching learning strategy.

Our word2Vec model was developed using a process similar to that of NLP. **Figure 2** depicts the full process of creating an EHR vector representation of a patient's clinical event. Starting the stemming system, we only retain the fluid-related blend name provided from input by the worldwide gathering computation. Individual reports, as well as the complete corpus, may be found under these titles. These items may be expressed through multiple English words; we treat each item as a single word. After forming the language, we looked for the word vector. Our word2Vec model was built using the CBOW computation on this corpus, so an event can be represented as a word vector. Word vector event representation can differentiate between types of events. It is impossible to make any distinctions between similar kinds of events. The piece, its embedding rate, and the assessments of the fundamental signals are quite similar to those that patients experience. Therefore, each event is represented as *e_i_* = *<⃗e_i_* total amount, rate, body temperature, pulse rate, respiration rate, blood pressure, where the total amount, rate, and all the representative vital signs represent a medical event.

Adding the term “vector” increases the number of boundaries in our model to a potentially unacceptable high level. Over-fitting, lengthy preparation time, and other complications may arise due to the model's numerous limits. We decided to use the layered reduction technique on the word vector so reducing the length of the event vector depiction would be possible by using Rule Part Examination (PCA) to keep the event vector’s similitudes and differences intact [32,33].

### Handling missing values

A major problem in the time series of clinical information is the lack of information and uneven examination, which impedes the use of a deep learning model for mortality risk assessment. As a result of the Bayesian and the measurably learning hypotheses, the Gaussian Cycle Relapse AI approach was developed. Its advantage is a high degree of prediction precision that may be achieved with a relatively small number of hyper boundaries.

The missing value and occasional examination issues are commonplace in EHRs over the long term. Gaussian Cycle is used to pre-process each tolerant EHR data. The probability circulation of Y* over provided X* may be predicted by Gaussian Cycle given a preparation set X, Y. As a result, it is frequently used to assign blame for “missing information”.

The characteristics of EHR information include missing attributes, large dimensionality, unexpected examining, and varied information types. These concerns might considerably impact the presentation of the expectation model. **Figure 3** depicts the patient record's missing value and unexpected inspection difficulties. Clinical records may lack certain features, thus inconsistently portraying the patient's course of events. A key problem in the clinical information time series is the lack of information and due to irregular sampling and sparse data. The commonly used approaches for missing observations are Pairwise Deletion (PD), Mean Substitution (MS), Regression Imputation (RI), Last observation carried forward (LOCF), Maximum Likelihood (ML), Expectation-Maximization (EM), and Multiple Imputation (MI). GP has been a leading and more efficient data imputation approach [35]. It utilizes the “kernel trick”; a kernel function measures the similarities between observed samples, then imputing the missing value with the maximum likelihood.

The perceptions are a persistent variable in the Likelihood and Gaussian hypothesis, for which the Gaussian interaction is an irregular cycle. Every aspect of a patient's health care record is expected to be constantly and time-sensitively irregular. The mean and portion work make up the bulk of the Gaussian Cycle.


1)

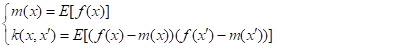



Here, *x, x′E R^d^* is the arbitrary random variable. Therefore, Gaussian Process can be defined as:

2) *f (x) ~ GP (m(x), k(x,x*′))

As for data set *{x, y}* it is possible to forecast its value u sing the following model for n observations:

3) *y = f (x) + ɛ*

The *x* is an input vector with the d dimension and *y* is the output vector. *ɛ* is the noise we added to the model by following the normal distribution, *ɛ ~ N(0,σ^2^_n_)*, its standard deviation is *σ^2^_n_*. The output of *y* satisfies the distribution:

4) *y ~ N(m(x), k(x,x) + σ^2^_n_  I_n_)*

Here, *I_n_* is the identity matrix. Pre-processing the data reduces its mean capacity to zero, and this is the default setting. Considering the concept of Gaussian Interaction, any constrained irregular elements operating together can also meet the Gaussian distribution. Let *x = {x*_1_, *x*_2_, *x*_3_, *..., x_n_}* be the collection of patient’s event occurring time-stamp sequence from the patient record with *n* number of events; we can denote it as *{f*(*x_i_*): ∈ *x_i_ x}*, which is the corresponding observed measurement value *y*, The predicted imputation value f_*_ and kernel function *k*( *· , ·* ). Then, the distribution of the set *x* is denoted as:


5)

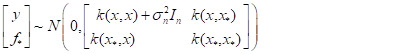



The *k(x,x)* is the covariance matrix with the dimension of n by n, matrix element *k_i,j_ = k(x_i_,x_j_), k(x,x_⁎_) = k(x_⁎_,x)^T^*  is the covariance matrix between the predicted imputation [INSERT Figure 003] and the training input vector *x* that has the dimensionality of n by 1. *k(x_⁎_,x_⁎_)* is the covariance of the predicted imputation *x_⁎_*. Hence, the posterior probability distribution *f_⁎_* and variance σ(*x_⁎_*) can be calculated as follows:


6)

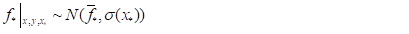




7)

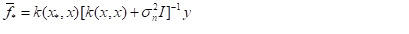




8)

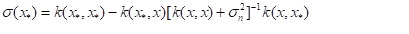



Where, the mean vector *f̅_⁎_* is the output of the Gaussian Process Regression model, so, the output value of the imputation point is:

9) *f_⁎_ = m*(*x*) + *f̅_⁎_*

For the choice of the kernel function, covariance work that has been squared outstanding (SE) has been selected as the recipe (Equation 8). There's a desire to shift from a Gaussian dispersion to a co-difference network while preserving the similarities between the two perceptions. The bit work must meet Mercer's requirements *k*(*x_i_*, *x_j_*) for a part modification (delineated in definition 1). The capacity must be square-integrable in Mercer's case (delineate in definition 2) As a result, the most commonly used bit work is the squared noteworthy piece, which is distinguished by its condition (equation 1), where parameter *σ^2^_f_* denotes the amplitude (y-scaling) and τ determines the smoothness of the Gaussian process prior with *k_SE_*( *· , ·* ).

Definition 1. A real-valued kernel function *k*(*x,y*) satisfies Mercer’s requirements if ∫  ∫ *K*(*x, y*)*g*(*x*)*g*(*y*)*dxdy≥*0 for all square-integrable functions g( · )

Definition 2. A function g(x) is square-integrable if



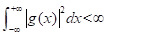




10)

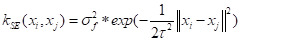



The squared exponential covariance function has only two hyperparameters, namely signal variance *σ^2^_f_* and length-scale τ. To find the most perfect characteristics, we used the most extreme probability strategy to deal with their fitting initials worth and then applied the Newton technique enhancement approach throughout the model preparation. First and foremost, we put up the negative logarithm probability calculation *L*(θ).


11)

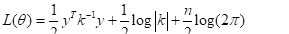



We found the derivative respective to the θ_i_


12)

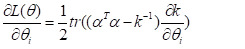



Where, *k = k_f_ + σ^2^_n_ I_n_*, **α** = *k^-^*^1^*y*. Equation 7 and 8 are utilized to generate the corresponding prediction value and standard deviation for the imputed value x, once we have found the most optimum parameters on the training data set. Then, if we want to impute the missing value *f*(*x_k_*), we need to calculate the new covariance matrix using the kernel function. We need to calculate the new vectors k*_SE_(*x_k_*, · ) and k*_SE_(*x_k_*, *x_k_*), where:


13)

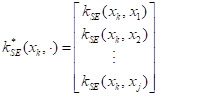



And 14) 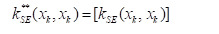


Then, the new co-variance matrix can be:


15)

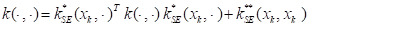



The value that is assigned to something might be the value that is most likely to be found using the new co-difference grid.

**Figure 4:**
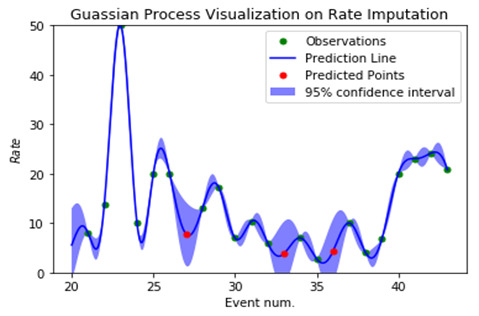
Gaussian Process effect visualization on Rate.

**Figure 5:**
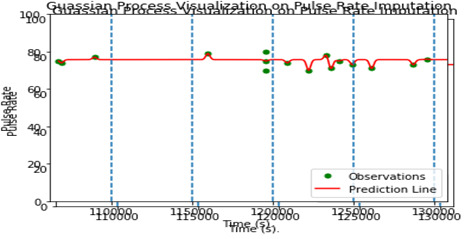
Gaussian Process effect visualization on Pulse Rate.

The missing-esteem issue on “rate,” “aggregate sum,” and all other vital sign estimates may be seen in the vector representation of the clinical event. The “rate,” “aggregate sum,” and their clinical events are distinct elements. We can effectively use the above Gaussian Interaction to process the greatest probability upsides of “rate,” “aggregate sum” for missing-esteem ascription in any clinical situation. All clinical occasion vectors, nevertheless, use their mean characteristics during the stretch with the premise that they should be consistently dispersed during the span for the assessment of essential signs. Indeed, all the important estimations are either strangely evaluated or completely lacking within the time period. We used Gaussian Cycle to standardize the timestamps when this basic estimation was recorded and approved, and we can now substitute the missing qualities for all critical sign estimations, making them regularly reviewed and working out the mean, as seen in **Figure 4** and **Figure 5**. An irregular example of Gaussian Interaction is depicted graphically in **Figure 4**.

The display of our pipeline necessitates the attribution of information. Because these two points of double cross are close in proximity, it is likely that the “rate” and “aggregate total” will have an influence on or be comparable at time *X_j_.* computing burden might possibly be heavy, especially if we need to build the detailed model. We intend to construct another Gaussian Interaction Regressor if one of the information highlight aspects is found to be unexpectedly inspecting or missing worth. Until now, every silence has been treated as a fixed-length arrangement of clinical events with assigned information. As a result of our innovative teaching approach, we expect this type of achievement to be a common occurrence. **Table 2** shows the superiority of GP over its competitors.

**Table 2:** Performance evaluation matrix for missing observation

Method	Precision of the method (Training data set)	Precision of the Method (Validation data set)
MI	0.7791	0.7411
PD	0.6950	0.6701
MS	0.8113	0.7931
GP	0.8312	0.8101
ML	0.7931	0.7313
EM	0.7804	0.7801
LOCP	0.7173	0.7305

MI – mutual information, PD – posterior distribution, MS –mean square, GP – Gaussian process, ML – machine learning, EM – early measure, LOCP – low order current position

GP for missing observations outperform for both trainings as well as validation data sets which are selected for implementation.

### Deep learning procedures

#### LSTM – RNN

Right present, RNN is the finest example of intermittent neural structure for sequential information. As a result, the patient's treatment contact is frequently interrupted by “disappearing inclination” issues and spams for long periods of time. For the problem of diminishing inclination, German researchers Hochreiter and Schmid-Huber [14] developed the LSTM repeating brain architecture called whose design can be viewed as a gated cell. When a door is opened and closed, the cell decides which data will be retrieved or forgotten. It is possible for LSTM to continue learning for a long period of time by following the door switch. Neurons in the LSTM have three types of entryways, namely information door, yield entryway, and avoidance entryway.

16) *i_t_ = σ_i_* (*W_xi_x_t_* + *W_hi_h_t-1_ + w_ci_ c_t-1_ + b_i_*)

17) *f_t_* = *σ_f_* (*W_xf_ x_t_ + W_hf_ h_t-1_ + W_cf_ c_t-1_ + b_f_*)

18) *c_t_ = f_t_ c_t-1_ + i_t_tanh* (*W_xc_x_t_ + W_hc_ h_t-1_ + b_c_*)

19) *o_t_ = σ_o_* (*W_xo_ x_t_ + W_ho_ h_t-1_ + w_co_ c_t-1_ + b_o_*)

20) *h_t_ = o_t_ tanh*(*c_t_*)

Where, *i_t_*, *f_t_*, *o_t_*, respectively represents the input gate, output gate, and forget gate function at time (t). *c_t_* is the activation vector, *x_t_* and *h_t_* are the input vector at time t and the hidden layer output vector at time t. *σ_i_*, *σ_f_*, *σ_h_*  are sigmoidal nonlinearities and, *σ_c_* and *σ_h_* are 10th non-linarites. *W_xi_*, *W_hi_*, *W_xf_*, *W_hf_*, *W_xc_*, *W_hc_*, *W_xo_*, *W_ho_* are the matrix parameters of the neural network. *W_ci_*, *W_cf_*, *W_co_*, *b_o_*, *b_i_*, *b_f_, b_c_* are the vector parameters of the neural network. Among them, *W_xi_*, *W_hi_*, *W_xf_*, *W_hf_*, *W_xo_*, *W_ho_* are the weight parameters for different gates, with bias *b_o_*, *b_i_*, *b_f_* being an element-wise (Hadamard) product. Since the LSTM decides to drop up some information at each time stamp, it is able to store the information from longer time stamp, when comparing with base-RNN.

### Phased-LSTM RNN

**Figure 6:**
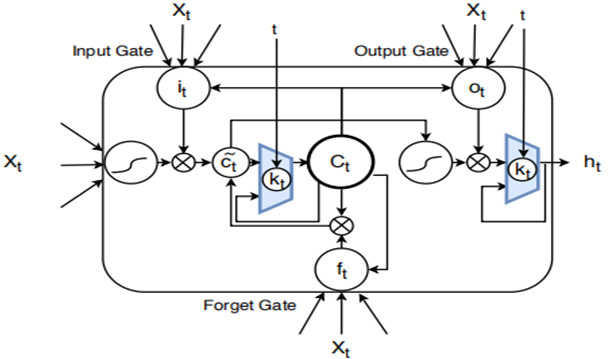
Depicts the Staged Long Short Term Memory (LSTM) cell's design.

Long-haul dependence is increasing; thus researchers are concentrating their efforts on developing a better LSTM cell. Using the LSTM as an example, in this study, each hidden layer neuron particularly staged LSTM has a period door (stage entrance). There is little doubt that the architecture of the neuron may be enhanced further; for example, by adding a channel door to display the model as presented in **Figure 6**. Staged LSTM [15] is a technique we use in addition to the standard LSTM to show the treatment results of patients. In contrast to ordinary LSTM, staged LSTM can cope with information that is only examined intermittently due to events that take place over a long period of time. Using staged LSTM may be more effective to demonstrate our worry since the information in the EHR may be triggered by unexpected events.

LSTM adds a new *kt* of time. When the doorway is opened, the modifications to neuron *c_t_* and *h_t_* must be made. Since the information may be reviewed at regular intervals, the issue of a too lengthy period can be addressed. Door opening and closing are restricted by free mood, which is dealt with through three boundaries involve determining the duration of a single opening and closing cycle. The stage duration is controlled by *r_on_* in proportion to the overall time frame, while *s* governs the movement of each cell in the cycle. During the training, students develop an understanding of these limitations. The equations can be found in following section.

20) *φ_t_ =* [(*t – s*) *mod τ*]/[τ]


21)

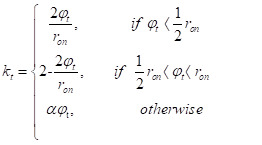



*ϕ_t_* demonstrates the many steps of the process. The opening of the door has two stages: the initial percentage grows from 0 to 1 in the first half of the opening, and then decreases from 1 to 0 in the last half. A spillage rate exists that can reveal considerable information when the entrance is closed; if this were not the case, no data would be stored in the hidden layer. With this time door, the Staged LSTM can address the issue of intermittent testing and speed up the training expression. For example, if the number of drug-related incidents in the present stretch is considerable, the initial proportion can be big (near 1); nevertheless, the initial proportion will acclimatize to a small value (near 0). For Staged LSTM cells, the value of the starting percentage and how much data are refreshed in the result layer and secret layer depend on how many drug events occur during a given h*_t_ time period. The calculation of *c_j_* and *h_j_* are performed based on equations 22-25, and the previous *c_t_* and *h_t_* will be denoted as c*_t_ and h*_t_.

22) c*_t_ = *f_t_ c_t-1_ + i_t_ tanh*(*W_xc_ x_t_ + W_hc_ h_t-1_ + b_c_*)

23) c_t_ = k_t_c*_t_ + (1 – *k_t_*)*c_t-1_

24) *h^2^_t_ = O_t_ tanh*(c*_t_)

25) h_t_ = k_t_ h*_t_ + 1 – *k_t_*)* *h_t-1_*

Then, we deﬁned the softmax layer that maps the outputs generated by the LSTM and Phased-LSTM cell into the probability representation using Equation (26), where *f(C_ti_)* denotes as the probability of class *i*.


26)

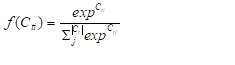



### Data and computational environment

#### Data

We conducted the experiments using Impersonate III's death hazard expectation and clinical implantation drug event hazard forecast data sets. Our data was divided into three sections: preparing (60%), approving (20%), and testing (10%) (20% of the all-out perception). A total of 46 520 patients are included in the Copy III database, which is a large quantity in terms of the number of patients hospitalized.

Liquid-related data are broken into three categories: 1) The term “itemid” refers to a drug or chemical that is liquid in nature. 2) “rate” records the pace at which the drug or substance was administered to the patient. 3) “aggregate sum” is used to record the total amount of liquid secured for the arrangement. There are two fields in the data table that keep track of when an information/yield event begins and ends. This information will be used to build a time-series of events for each individual patient.

The following is a breakdown of the most accurate calculations for essential sign records: First, unusual levels of internal heat may be caused by fever, hypothermia, or any drug that has an adverse effect. Second, heartbeat rate can be included as a heart musicality and the power of the beat. An unusual breath rate might be caused by a fever or other illness. There are four stages to the pulse, which reflect the status of the heart: normal, elevated, stage 1, and stage 2. The “rate,” “aggregate sum,” and all the following estimations are constant mathematical characteristics that experience the absent qualities and unexpected investigating.

## RESULTS

### Structure of deep learning

**Figure 7:**
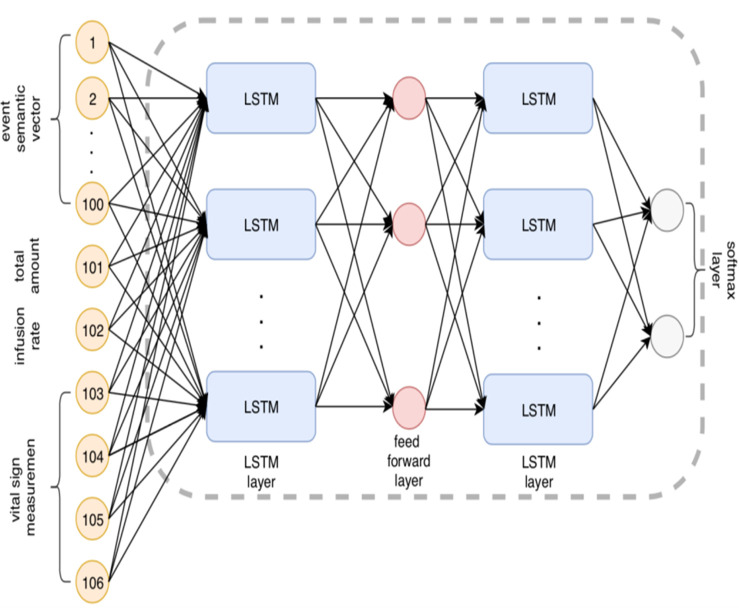
The structure of Long Short Term Memory (LSTM) based model.

**Figure 8:**
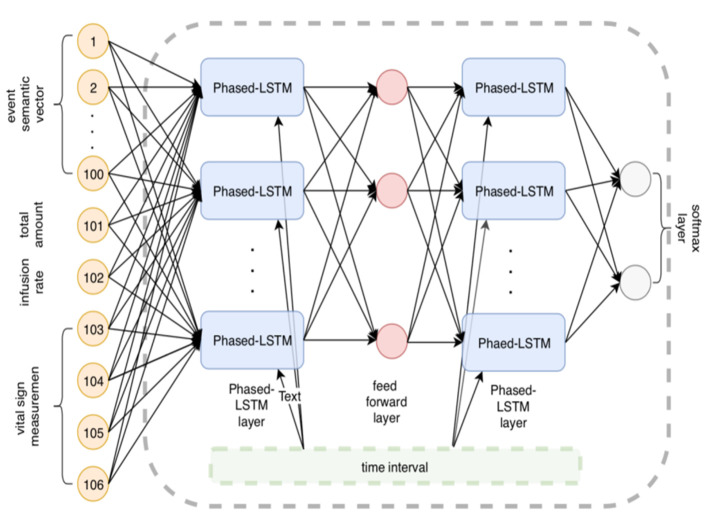
The structure of improved Phased-Long Short Term Memory (LSTM) based model.

**Figure 7** and **Figure 8** show the LSTM and Staged LSTM models we built, which have a similar architecture. There are 105 inputs and 310 hidden units with two levels. The user decides how many units, elements, and layers are stored away. We add a softmax layer towards the end of the organizing process to help with the characterization process. To use LSTM for deep neuronal organization, the user must add a last piece of information: time. LSTM-based models and staged LSTM models were produced using the clinical events we scheduled according to the client's request for their occurrence times. We used the R-bundle “rnn” to run the LSTM model and Staged LSTM on it.

Even though our models and data set are assigned, the outcome of our experiments is not predetermined. Capabilities and boosters for bad luck were discussed. When using AMO as a streamlining agent, the model produced the most accurate results.

There are two probabilities for the model output: [*Prob(survival), Prob(dead)*], denoted as [*p(v_1_), p(v_2_)*] or *p(v_1_)>p(v_2_)*; in case of the latter, we classify the patient as dead (0), otherwise, we classify the patient as surviving (1).

The Matthews correlation coefficient (MCC) and the receiver operating characteristic curve (ROC) were used to assess the capacity to distinguish between survivors and non-survivors (ROC). In cases where the patient survives, we assign a score of zero, which means that *p(v1)>p(v2)*. If the patient outcome is death we classify it as one, then the target variable is (0, 1), and the interpretation is that *p(v1)<p(v2)*. We saw the challenge as a binary classification issue. When using binary classification, the Mathews correlation coefficient (MCC) is defined to be



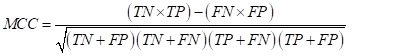



There are no obvious negatives or positives in the TP or TN models; only false negatives and false positives are depicted in the FN and FP models. The Pearson connection coefficient discretized for double components [37] shows that MCC moves from 1 (all cases were correctly predicted) to 1 as a prevalence measure for parallel order (all examples mistakenly anticipated). MCC is regarded as an acceptable metric that provides value for data sets that are unbalanced [38-39].

**Figure 9:**
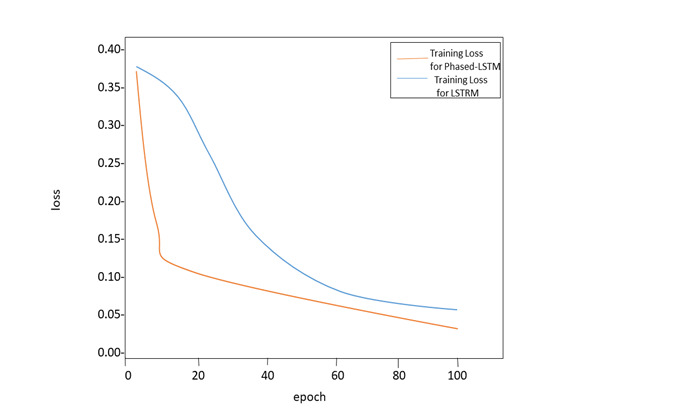
The training loss of Long Short Term Memory (LSTM) and Improved Phased-LSTM: epoch = 100, learning rate = 0.01.

At each epoch, the Phased-LSTM and Improved-LSTM lose their ability to learn. The RNN and LSTM training phases are defined by the epoch. The MSE mathematical function calculates the model's loss, which represents the model's earning results. Both models are designed to minimize the amount of training value that is lost. Initial LSTM losses are smaller than Phased-LSTM losses. However. after 10 epochs, the Phased-LSTM losses can drastically diminish the value of LSTM losses (converge at a faster rate). The Phased-LSTM itself is to blame for this. As a result, information cannot enter the cell's memory when the Phased-LSTM time gate is closed or opened, resulting in a higher loss of Phased-LSTM. The Phased-LSTM, on the other hand, may converge at an astonishing rate, reducing its loss quickly throughout the training phase (**Figure 9**).

### Performance evaluation

We initially explore the effect of using Gaussian Interaction for information ascription on the two models’ presentation. Then, we consider about the outcomes gained by LSTM model and Staged LSTM. Finally, we contrast the profound learning models and other AI computations. To illustrate that the Staged LSTM manages to down the long succession information, we sift through patients with few clinical occasions and generate the preparation, approval, and testing data sets with the suitable quantity of patient occurrences.

**Figure 10:**
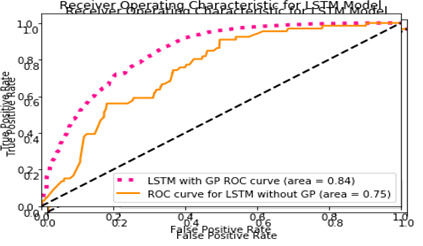
The Receiver Operating Characteristic (ROC) curve with application of Gaussian Process for Long Short Term Memory (LSTM) model.

**Figure 11:**
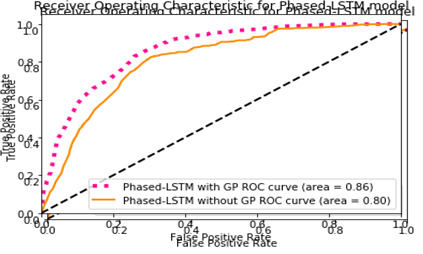
The Receiver Operating Characteristic (ROC) curve with application of Gaussian Process for Phased-Long Short Term Memory (LSTM) – model.

We employ ROC bend, accuracy, and review score to judge the model's exhibition. Execution assessment of the proposed method with and without missing characteristics procedure (like GP) is presented in **Table 3** underneath. Receiver Operating Characteristic (ROC) curve of the proposed techniques is displayed in **Figure 10** and **Figure 11**.

**Table 3:** Performance evaluation of the proposed method with its competitors – SAPS-II, APS-III, SOFA, base LSTM and Phased-LSTM

Method	Precision		Recall	
**Without (Induction of missing values technique)**	**With (Induction of missing values technique)**	**Without (Induction of missing values technique)**	**With (Induction of missing values technique)**
SAPS-II	0.7614	0.7903	0.6831	0.7209
APS-III	0.7301	0.7549	0.6131	0.6701
SOFA	0.6941	-	0.6272	-
LSTM	0.8004	0.8571	0.7101	0.8322
Phased-LSTM	0.8287	0.8739	0.7838	0.8568

SAPS-II – Simplified Acute Physiology Score, APS-III – Acute Physiology Score, SOFA – Sequential Organ Failure Assessment, LSTM – Long Short Term Memory, Phased-LSTM – Phased Long Short Term Memory

**Figure 10****,** **Figure 11****,** and **Table 3** show that the advanced models outperform the initial Staged LSTM approach. The models with Gaussian Interaction have better accuracy, review ratings, and the ROC curve bends. This is because the medical services data set has a substantial number of missing characteristics. Unless we assign them, the model's presentation will be impaired. Notably, Gaussian Interaction models have a second problem: the amount of preparation time. We want to construct a GP regressor for each example, approve it, and then contribute to the model for each case. As predicted, the Staged LSTM model is also ready to deliver superior results, given that every tolerance example mostly has an extended consecutive dependency.

## CONCLUSIONS

It is difficult to examine medical services information in an ICU environment because of the lack of quality, occasional testing, various information kinds, high dimensionality, and long duration of dependency on the information. We came up with a strategy for preparing development based on a factual approach and word insertion. To deal with the unexpected testing and extended successive dependency inside the data set, we offered another type of LSTM-RNN termed improved Staged LSTM. For the mortality expectancy problem, the results show that a staged LSTM deep learning system, coupled with the pre-processing pipeline described here, may produce promising results.

Rather than focusing solely on liquid-related clinical events, we plan to extend our pipeline and model to other complex data sets in the future. Additional information on vitamins, drugs, and other devices will be added to the board soon. In theory, the model more accurately forecasts risks, analyses clinical prescription opportunities, and automates the administration of major hardware more than previously thought possible. Future research might focus on improving the suggested approach's ability to accurately predict the outcomes of real data sets.
